# EMD-Based Energy Spectrum Entropy Distribution Signal Detection Methods for Marine Mammal Vocalizations

**DOI:** 10.3390/s23125416

**Published:** 2023-06-07

**Authors:** Chai-Sheng Wen, Chin-Feng Lin, Shun-Hsyung Chang

**Affiliations:** 1 Department of Electrical Engineering, National Taiwan Ocean University, Keelung 20224, Taiwan; 21053001@mail.ntou.edu.tw; 2 Department of Microelectronics Engineering, National Kaohsiung University of Science and Technology, Kaohsiung 81157, Taiwan

**Keywords:** detection, empirical mode decomposition, energy spectrum entropy, receiver operating characteristics

## Abstract

To develop a passive acoustic monitoring system for diversity detection and thereby adapt to the challenges of a complex marine environment, this study harnesses the advantages of empirical mode decomposition in analyzing nonstationary signals and introduces energy characteristics analysis and entropy of information theory to detect marine mammal vocalizations. The proposed detection algorithm has five main steps: sampling, energy characteristics analysis, marginal frequency distribution, feature extraction, and detection, which involve four signal feature extraction and analysis algorithms: energy ratio distribution (ERD), energy spectrum distribution (ESD), energy spectrum entropy distribution (ESED), and concentrated energy spectrum entropy distribution (CESED). In an experiment on 500 sampled signals (blue whale vocalizations), in the competent intrinsic mode function (IMF2) signal feature extraction function distribution of ERD, ESD, ESED, and CESED, the areas under the curves (AUCs) of the receiver operating characteristic (ROC) curves were 0.4621, 0.6162, 0.3894, and 0.8979, respectively; the Accuracy scores were 49.90%, 60.40%, 47.50%, and 80.84%, respectively; the Precision scores were 31.19%, 44.89%, 29.44%, and 68.20%, respectively; the Recall scores were 42.83%, 57.71%, 36.00%, and 84.57%, respectively; and the F1 scores were 37.41%, 50.50%, 32.39%, and 75.51%, respectively, based on the threshold of the optimal estimated results. It is clear that the CESED detector outperforms the other three detectors in signal detection and achieves efficient sound detection of marine mammals.

## 1. Introduction

Radio waves and light waves in seawater propagation undergo significant attenuation; therefore, they cannot convey information reliably. Sound waves are currently known to be the most effective long-distance carriers of information in the seawater medium. Their underwater speed is four times faster than that in air. Therefore, in the dark environment of the deep sea, marine mammals often rely on vocal communication, making the ocean replete with vocal sounds, clicks, pulses, whistles, moans, and other evocative melodies and songs [1]. The research and development of underwater acoustic technology includes the acoustic characteristics of the seawater medium [2], the propagation characteristics of sound waves in the seawater medium, and the sound characteristics of underwater targets. Underwater signal processing is a very important part of the process of studying these sound characteristics; it includes sound recording and preprocessing and signal feature extraction, detection, and classification [3,4]. The sound of marine mammals can be recorded using a hydrophone and recording equipment. The current sonar systems are of two types, active and passive, and are implemented through technologies such as towed array sonars, sonobuoys, and bottom-mounted sensors. Further, a passive acoustic monitoring system can be used to record a large number of raw underwater sound signals [5] which may contain marine mammal sounds, ship engine sounds, sounds of lapping water, and unknown noise. Most of these underwater sound signals are nonstationary signals. Therefore, the raw underwater sound signals need to be processed first. These raw signals are effectively sampled, signal feature extraction and analysis are performed on the sampled signals, and feature extraction is used to extract the useful signal characteristics to improve the accuracy of the detector and classifier by removing redundant data [6].

Over the years, various techniques have been developed for feature extraction, detection, and classification of cetacean signals, such as short time Fourier transform (STFT). Gillespie et al. [7] proposed a two-stage process for a detector, wherein the spectrogram is smoothed by convolving it with a Gaussian kernel and the outlines of the sounds are extracted using an edge detection algorithm. Lopatka et al. [8] presented some of the advantages of the wavelet transform (WT) and spectrogram in analyzing sperm whale clicks and proposed a new parameter called short-time window energy for detection. Alam et al. [9] compared three time–frequency representations: the Fourier Transform, the wavelet transform, and the Hilbert–Huang transform (HHT). Based on the results, HHT proved to be a viable substitute for WT. Liu et al. [10] applied the instantaneous harmonic retrieval method to calculate the instantaneous frequencies of the intrinsic mode functions (IMFs), proving that the improved version is effective for underwater acoustic signal detection. Seger et al. [11] proposed an empirical mode decomposition (EMD) detection and classification process to extract possible signals from a dataset with minimal postprocessing quality control. Mazhar et al. [12] used feature extraction for the recognition of individual humpback whale vocalizations. Pace et al. [13] presented three feature extraction methods (cepstral, linear prediction, and mel-scale frequency cepstral coefficients (MFCCs)) to extract the characteristics of humpback whale vocalizations. The detection stage is the process of identifying the target marine mammal signals among other unwanted signals that may exist. Most detection and classification algorithms use signal attributes to develop feature extraction methods and capture different feature parameters of the target signal. However, they attempt to generate the best profile of the desired signal according to the current target and environmental conditions and observe the series of characteristic parameters. Murray et al. [14] reported a self-organizing neural network (NN) for categorizing the repertoire of false killer whale vocalizations. In addition, it is worth noting that neural networks have made significant advancements and have demonstrated remarkable performance in the field of underwater sound classification [15,16]. Ibrahim et al. [17] used MFCCs and discrete WT to extract the features of North Atlantic right whale up-calls and proposed a new up-call detection algorithm and classifiers, such as support vector machines (SVMs), which can be applied to classify the call types. Statistical-based detection and classification techniques, such as Gaussian mixture models, hidden Markov models, NNs, and SVMs, use statistical inference to discover the best patterns for matching signal features. In addition, in threshold-based technologies, detection and classification techniques are set for the model based on a defined threshold. The model then searches for correlations between the dataset structure and the known templates. The adaptive setting of the optimal threshold and the recording of the detection results when the threshold is exceeded or unattained have improved signal detection accuracy. Altes et al. [18] presented a locally optimal detector that correlated spectrogram data with maximum-likelihood parameter estimation. Bouffaut et al. [19] proposed a new method based on the passive application of a stochastic matched filter to detect Antarctic blue whale Z-calls in noisy underwater environments. Erbe et al. [20] proposed a new detection method using the entropy of information theory (Shannon) which detects the calls of a variety of cetaceans and surpasses the performance of two commonly used detectors based on peak energy detection and multi-band energy detection. Permutation entropy and sample entropy (SE) are measures of complexity that have been used as metrics for the unattended detection of whistles and clicks in passive acoustic monitoring (PAM) data. Siddagangaiaha et al. [21] proposed these metrics and tested their detection capabilities by applying them to PAM data from two study sites: Eastern Taiwan Strait in Taiwan and Honolua Bay in Hawaii, United States.

The use of time–frequency distributions (TFDs) [22], the short-time Fourier transform (STFT), the Wigner–Ville distribution [23], and WT [24] to achieve more accurate signal resolutions for nonstationary and nonlinear signals is an interesting area of research. At present, most of the TFD functions, based on the kernel window, have the advantage that the spectrum of the signal can be easily and quickly parsed; however, these methods are often limited by the length of the sampling points of each function conversion time, resulting in a decrease in the instantaneous frequency (IF) resolution, and it is impossible to accurately analyze the time and frequency characteristics. HHT [25], proposed by Huang in 1996, is a mathematical tool used for analyzing signals, particularly in the field of signal processing. It is a linear operator that can be applied to a wide range of signals, including nonstationary and nonlinear signals, which applies the sifting process of EMD. (The EMD method decomposes a signal into a set of IMFs and a residual function (RF).) The HHT algorithm is used to provide a high-frequency resolution analysis of a signal, which is achieved by analyzing the signal at the IMF level. Each IMF has a distinct frequency range, and the HT is used to extract the IF of each IMF, allowing for a detailed analysis of the signal’s frequency content over time.

**Figure 1:**
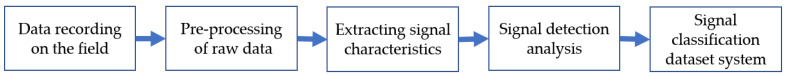
The five main areas of research on marine mammal vocalization.

To develop a diverse passive acoustic monitoring (PAM) system capable of adapting to the challenges of complex underwater environments, research on marine mammal sounds can be divided into the following main areas: signal data recording in the field, preprocessing of raw data, extraction of signal characteristics, signal detection analysis, and signal classification dataset systems (Figure 1). 

Firstly, field signal data recording involves collecting raw data in underwater environments. Subsequently, preprocessing steps, such as filtering, noise reduction, and interference removal, are necessary to enhance the quality and distinguishability of the signals. Next, feature extraction is a crucial step that involves extracting key characteristics of cetacean sounds from complex underwater signals. The feature extraction algorithms need to capture energy characteristics, spectral properties, and other relevant information of cetacean sounds. Such feature extraction algorithms assist in identifying and distinguishing cetacean sounds from environmental noise and interference. Additionally, signal detection analysis and the deployment of signal classification dataset systems are essential steps. Detection algorithms should effectively detect cetacean sounds and differentiate them from other types of signals. This differentiation aids in further signal analysis and subsequent classification of cetacean sounds. Therefore, we have developed efficient feature extraction and detection algorithms. These algorithms can extract and analyze key features of cetacean sounds, enabling accurate detection. This has significant implications for cetacean research, marine ecosystem conservation, and environmental management. 

In this study, we successfully developed the energy characteristics analysis methods proposed in previous research [26,27] by harnessing the advantages of the empirical mode decomposition (EMD) method to parse the sound signals of multiple marine mammals. The sound signals were transferred from the time domain to the frequency domain using the marginal frequency (MF) method, and the changes in signal energy were obtained. It can be found that different marine mammal species emit a variety of behavioral sounds with unique energy characteristics. Therefore, we introduced the concept of the average number of data with entropy in information theory [28], where the higher the entropy, the greater the amount of information that can be transmitted, and vice versa. In other words, we can calculate the entropy to determine the amount of information contained in the signal and the degree of its change. When the information is more certain and specific, the entropy is lower, and when it is more uncertain and confusing, the entropy is higher. Then, according to the total signal energy or main frequency domain of energy concentration, the energy distribution function of each sampling frequency in the spectrum is normalized. The energy distribution function of each sampling signal can be represented by a probability function, and after entering the calculation formula for entropy, the entropy of the energy spectrum in each sampling signal can be obtained, which is called the energy spectrum entropy. Four signal feature extraction and analysis algorithms are proposed in this paper: energy ratio distribution (ERD), energy spectrum distribution (ESD), energy spectrum entropy distribution (ESED), and concentrated energy spectrum entropy distribution (CESED). The time–energy distributions or energy spectrum entropy distributions generated by these algorithms are observed, and threshold-based technologies of detection theory are used in the signal feature extraction function distribution of each algorithm to successfully and efficiently realize sound detection of marine mammals.

The remainder of the paper is organized as follows. In Section 2, the proposed detection algorithm with five main steps and four signal feature extraction and analysis algorithms are presented. The sampling process, energy characteristics analysis, MF distributions, feature extraction, and signal detection are described. In Section 3, the receiver operating characteristic (ROC) curves of ERD, ESD, ESED, and CESED with the optimal estimated threshold are presented. In Section 4, the signal feature extraction function distributions of ERD, ESD, ESED, and CESED for the areas under the curves (AUCs) and detection accuracy with the optimal estimated threshold are discussed. In addition, the ROCs of ERD, ESD, ESED, and CESED for the sound of the bowhead whale and the Bryde’s whale are presented. Finally, Section 5 concludes the paper.

## 2. Method

**Figure 2:**
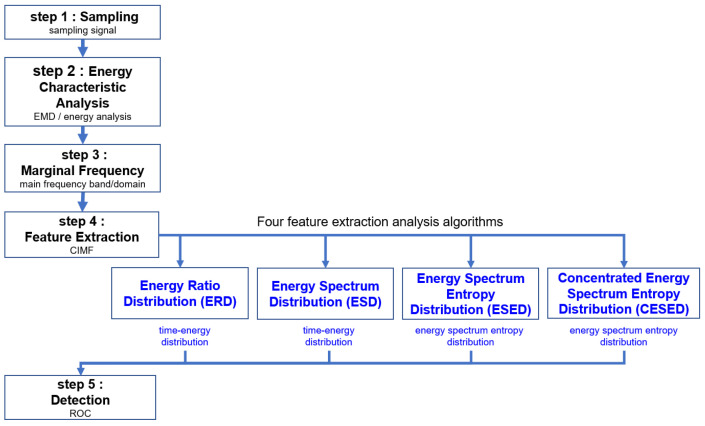
Proposed detection algorithm with five main steps and four signal feature extraction and analysis algorithms.

Based on the EMD method, four signal feature extraction and analysis algorithms are proposed, namely, ERD, ESD, ESED, CESED, and there are five main steps: sampling, energy characteristics analysis, MF distribution, feature extraction, and detection, as shown in Figure 2. The proposed method was applied in the field of marine mammal vocalization signal detection. 

### 2.1. Sampling

**Figure 3:**
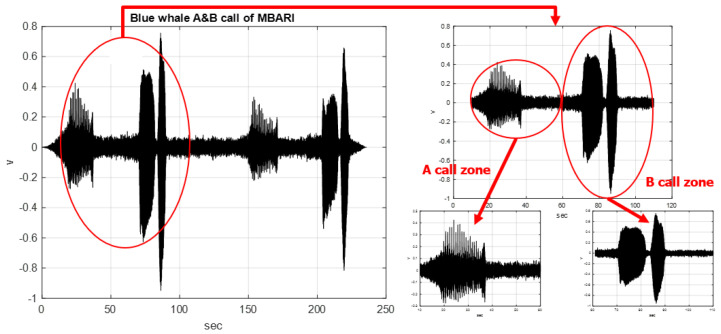
Two sets of A and B call vocalizations of blue whales (duration: 235.72 s; sampling frequency: 4800 Hz) and one set of valid sampling signals (duration: 100 s; 500 sampling signals) that included A and B call zones.

The marine mammal sound signals used in this study were obtained from the website of the Monterey Bay Aquarium Research Institute (MBARI), Moss Landing, CA, USA [29]. Figure 3 shows the A and B calls of two sets of blue whales with a sampling time of 235.72 s and a sampling frequency of 4800 Hz. One set of signals, A and B, belonging to the blue whales were captured as valid sampling signals during the experimental process of this study. The sampling time for this valid signal sampling was 100 s, 500 sampled signals were divided evenly, and each sampling signal lasted 200 ms. It can be observed that the first half of this valid signal sample contained 250 sampling signals for the A call zone of 50 s and that the second half contained 250 sampling signals for the B call zone of 50 s.

### 2.2. Energy Characteristics Analysis

**Figure 4:**
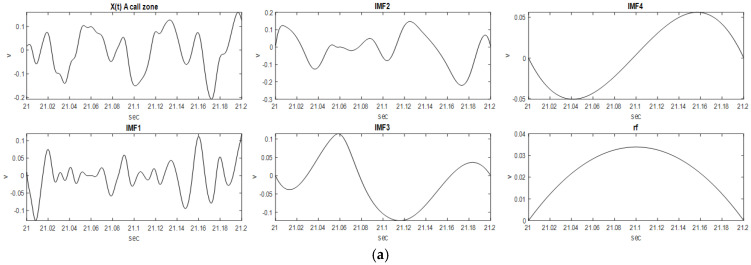

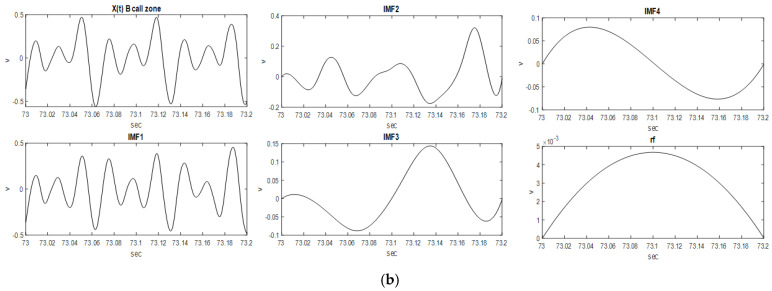
(**a**). One of the 500 sampling signals (blue whale vocalization): A call zone (20~20.2 s) with 4 IMFs and 1 *rf.* (**b**). One of the 500 sampling signals (blue whale vocalization): B call zone (73~73.2 s) with 4 IMFs and 1 *rf*.

EMD was used as the basic theoretical framework for the energy characteristics analysis of the IMF and *rf* of the sampling signal. The sampling signal X(t) can be decomposed into *N* IMFs and one *rf* after the shifting process of EMD.

(1) $$X\left(t\right)=\sum_{i=1}^{N}{IMF}_{i}\left(t\right)+rf\left(t\right)$$

where IMF*i*(t) is the *i*th IMF and *rf* is the residual function for the 500 sampling signals. The EMD for each sampling signal can obtain 21 sampling signals with 4 IMF*i*s (3 IMFs and 1 *rf*), 185 sampling signals with 5 IMF*i*s (4 IMFs and 1 *rf*), 254 sampling signals with 6 IMF*i*s (5 IMFs and 1 *rf*), and 40 sampling signals with 7 IMF*i*s (6 IMFs and 1 *rf*); two of the sampling signals are shown in Figure 4.

The total signal energy, E*_total_*, can be defined as the sum of the energies of all the IMFs. This is because the energy of the original signal is distributed among the different IMFs, and adding up the energies of all the IMFs gives the total energy of the original signal.

(2) $$E_{total}=\sum_{i=1}^{N}{IMF}_{i}^{2}\left(t\right)+{rf}^{2}\left(t\right)$$

The *i*th IMF energy ratio is:

(3) $$E_{IMFi}=\frac{{IMF}_{i}^{2}}{E_{total}}\;*\;100\%$$

As shown above, the average energy ratio of each IMF*i* can be calculated for 500 sampling signals, and the IMF*i* with the higher average energy ratio is defined as the competent IMF (CIMF) among the 500 sampling signals. The average energy ratios of IMF1, -2, -3, -4, -5, -6, and -7 are 31.92%, 25.09%, 19.29%, 12.14%, 6.62%, 1.90%, and 0.21%, respectively, as shown in Table 1. The average energy intensity densities are mainly concentrated in IMF1, -2, -3, and -4; the top two, IMF1 and IMF2, with high average energy ratios are taken as the CIMFs, which are used as the IMFs for the main signal analysis of the algorithm. 

### 2.3. Marginal Frequency

According to the theory of the TFD function, after implementing HT for each IMF*i*, the sampling signal can be expressed as the sum of the real and imaginary parts, and the IF of the *i*th IMF, Fi(t), can be calculated as:

(4) $$X_{i}\left(t\right)={IMF}_{i}\left(t\right)+jHT\left\{{IMF}_{i}\left(t\right)\right\}=A_{i}\left(t\right)e^{j\theta_{i}\left(t\right)}$$

(5) $$F_{i}\left(t\right)=\frac{1}{2\pi }\frac{d\theta_{i}\left(t\right)}{dt}$$

The sampling frequency bandwidth is *f* Hz, and the MF distribution (MF; frequency–energy distribution) of the *i*th IMF is defined as:

(6) $${MF}_{i}=\frac{{IMF}_{if}^{2}\left(t\right)}{E_{total}}\;*\;100\%$$

**Figure 5:**
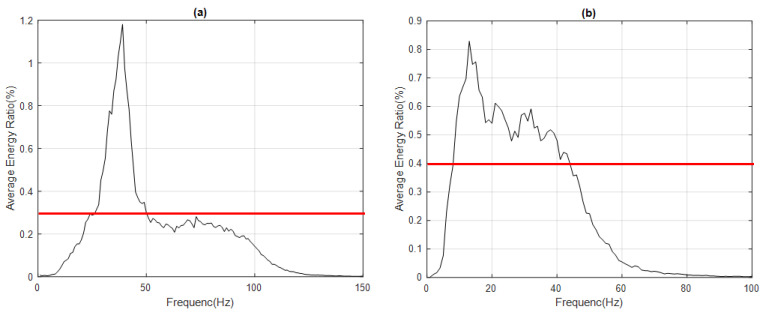
Average MF distributions of (**a**) IMF1 with threshold 0.3 (red line) and (**b**) IMF2 for 500 sampling signals with threshold 0.4 (red line) (blue whale vocalization).

Thus, the average MF distributions of IMF1 and IMF2 for 500 sampling signals were obtained, as shown in Figure 5.

Further, the appropriate threshold was selected. As shown in Table 2, when the threshold was 0.3, the main frequency band of IMF1 was 27–49 Hz, the frequency of the highest energy ratio was 39 Hz, and the main frequency domain was 1–150 Hz; when the threshold was 0.4, the main frequency band of IMF2 was 9–43 Hz, the frequency of the highest energy ratio was 13 Hz, and the main frequency domain was 1–100 Hz. To observe the tendency of the MF spectrogram, the energy of the A call and B call signals was mainly distributed around the frequency of the highest energy ratio for IMF1 and IMF2, respectively. 

### 2.4. Feature Extraction

After the sampling signals were subjected to EMD and energy characteristics analysis, the four signal feature extraction and analysis algorithms proposed in this paper, ERD, ESD, ESED, and CESED, which served as the theoretical basis for signal detection, were applied. Since the CIMFs had a higher average energy ratio among all IMF*i*s of the sampling signals and the energy signature component of the signal was distinct, the CIMFs and higher-energy IMF*i*s were taken as the main signals in the detection process.

#### 2.4.1. Energy Ratio Distribution (ERD)

**Figure 6:**
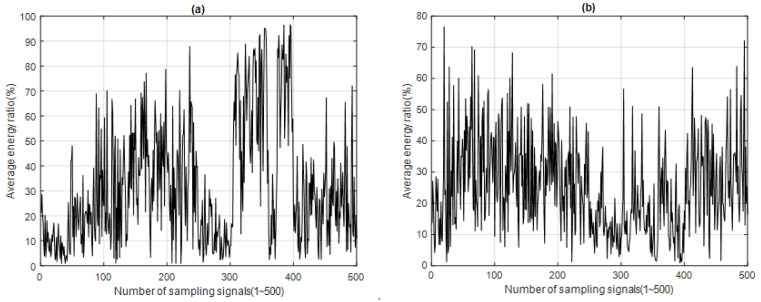
Time–energy distribution of (**a**) IMF1 and (**b**) IMF2 for 500 sampling signals (blue whale vocalization) using ERD.

The energy ratio E*_IMFi_*(*t*) of the IMF*i* of a sampling signal was calculated according to Equation (3) in the energy characteristics analysis, and then the time–energy distribution of each IMF*i* was obtained from the 500 sampling signals, as shown in Figure 6, for IMF1 and IMF2. The CIMF with the highest energy distribution ratio was IMF1, and the energy distributions of the two groups can be observed in Figure 6a, which individually fall in the A and B call zone. The A and B calls can be analyzed using IMF1. Figure 6b shows the energy distribution ratio of IMF2. It can be seen that there is an energy distribution in the A call zone, but the signal energy is relatively weaker in the B call zone. 

#### 2.4.2. Energy Spectrum Distribution (ESD)

From Equation (6), for the MF, the MF distribution of the *i*th IMF of the sampling signal was calculated, and the MF frequency–energy distribution of each sampling signal was obtained. Then, in the main frequency domain of the MF of each sampling signal, the energy of all instantaneous frequencies was scanned, and the frequency of the highest energy ratio Max(E*_IMFi_*(*f*)) was obtained.

(7) $$max(E_{IMFi}(f))=max\left\{{MF}_{i}(f)|f=f_{1}\sim f_{2}\right\}$$

**Figure 7:**
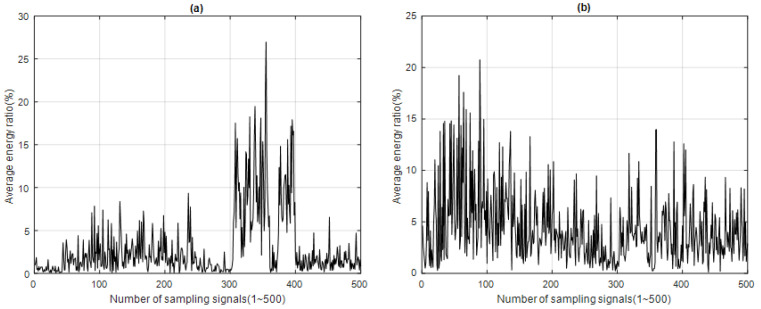
Time–energy distribution of (**a**) IMF1 and (**b**) IMF2 for 500 sampling signals (blue whale vocalization) using ESD.

*f*: *f*_1_*~f*_2_ is the main frequency domain of the MF of the sampling signal. The time–energy distribution of each IMF*i* can be obtained for 500 sampled signals, as shown in Figure 7 for IMF1 and IMF2, which are the energy ratios of the IF with the highest energy in the MF. The energy distributions of the A call and B call can be clearly observed in Figure 7a for IMF1, but from Figure 7b it is almost impossible to identify the energy distribution of the B call for IMF2.

#### 2.4.3. Energy Spectrum Entropy Distribution (ESED)

For each IMF*i*, the IF can be calculated using the HT, which provides the time-frequency–energy distribution, called the Hilbert energy spectrum.

(8) $${HS}_{i}=\frac{E_{i}\left(t,f\right)}{E_{total}}=\frac{{IMF}_{i}^{2}\left(t,f\right)}{E_{total}}$$

The energy distribution function E*_i_*(t, f) contains the sampling time (*t*) and sampling frequency (*f*), where the energy distribution function of each sampling signal defines E*_ij_* ∈ {E*_im_*, …, E*_in_*}. Here, *i* is the IMF number of the sampling signal and j is the sampling frequency range from *m* to *n*. Then, the energy distribution function is normalized according to the total energy of the signal, and the energy distribution function of each sampling signal can be expressed by the probability functions P*_ij_* ∈ {P*_im_*, …, P*_in_*}, which can indicate the signal energy density of the *i*th IMF in the sampling signal.

(9) $$P_{i}\left(E_{j}\right)=P\left(E_{ij}\right)=\frac{E_{ij}}{E_{total}}$$

where *i* is the *i*th IMF of the sampling signal and *j* is the sampling range from *m* to *n*. Since entropy can be used to measure the information uncertainty and is proportional to the amount of uncertainty in the data, the greater the uncertainty, the greater the entropy. Therefore, the entropy of the energy spectrum of each sampling signal can be calculated, which is called the ESED. In information theory, the unit of entropy depends on the logarithmic base used, with bits being the most commonly used unit. When the natural logarithm (base e) is used, the unit of entropy is nats.

(10) $$ESED=H\left(E\right)=-\sum_{i=1}^{i}\sum_{j=m}^{n}P\left(E_{ij}\right)\log P\left(E_{ij}\right)$$

The energy spectrum entropy (H) of each IMF*i* can also be determined.

(11) $$ESED\;of\;ith\;IMF=H_{i}\left(E\right)=-\sum_{j=m}^{n}P_{i}(E_{j})\log P_{i}(E_{j})$$

**Figure 8:**
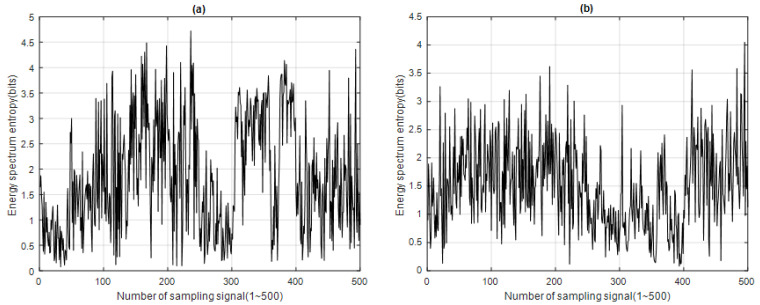
Energy spectrum entropy distribution of (**a**) IMF1 and (**b**) IMF2 for 500 sampling signals (blue whale vocalization) using ESED.

We obtained the ESED of each IMF*i* for 500 sampling signals, as shown in Figure 8.

In the MF process, the main frequency domain of the sampling signal can be determined, and most of the energy of the sampling signal will be concentrated in the main frequency domain. If the main frequency domain is equal to the sampling frequency range, the energy spectrum entropy of each IMF*i* in the main frequency domain, called H*_icd_*, can be calculated, and the main frequency domain ranges from *c* to *d*.

(12) $$H_{icd}\left(E\right)=-\sum_{j=c}^{d}P_{i}(E_{j})\log P_{i}(E_{j})$$

#### 2.4.4. Concentrated Energy Spectrum Entropy Distribution (CESED)

Since E*_total_* is the total energy of the sampling signal in the ESED, the frequency distribution is in the full frequency domain. However, when calculating the entropy in the ESED process, only the sampling frequency range is calculated. Hence, the calculated entropy may deviate from the real value. In addition, from the MF process, it is known that the energy of the sampling signal will be concentrated in the main frequency domain. Therefore, to increase the integrity of the sampling signal analysis, the energy distribution function is normalized in the main frequency domain of the sampling signal so that the best and worst values of each variable are adjusted between 0 and 1, and the energy distribution function of each sampling signal is redefined for E*_ij_* ∈ {E*_ia_*, …, E*_ib_*}, where *i* is the IMF number of the sampling signals and j is the main frequency domain from *a* to *b*. The maximum energy distribution function is *max*(E*_i_*), *max*(E*_i_*) = *max*{E*_ia_*, …, E*_ib_*}, and the smallest energy distribution function is *min*(E*_i_*), *min*(E*_i_*) = *min*{E*_ia_*, …, E*_ib_*}. The energy distribution function of each sampling signal can be expressed by the probability functions P*_ij_* ∈ {P*_ia_*, …, P*_ib_*}, which can also provide the signal energy density of the *i*th IMF in the sampling signal.

(13) $$S_{ij}=\frac{E_{ij}-\min\left(E_{ij}\right)}{\max\left(E_{ij}\right)-\min\left(E_{ij}\right)}$$

(14) $$P_{i}\left(E_{j}\right)=P\left(E_{ij}\right)=\frac{S_{ij}}{\sum_{j=a}^{b}S_{ij}}$$

Thus, the CESED of each IMF*i* can be calculated as follows:

(15) $$CESED\;of\;the\;ith\;IMF={CH}_{i}\left(E\right)=-\sum_{j=a}^{b}P_{i}(E_{j})\log P_{i}(E_{j})$$

**Figure 9:**
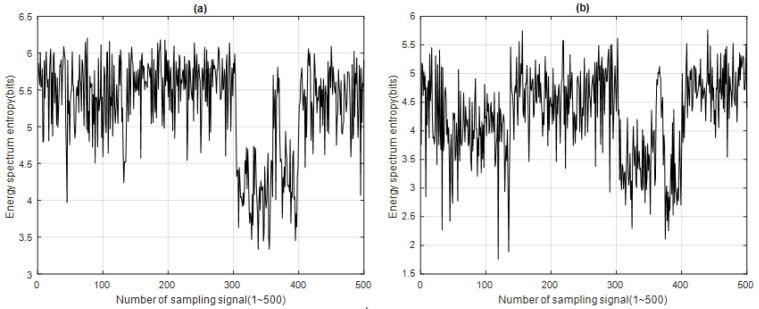
Concentrated energy spectrum entropy distribution of (**a**) IMF1 and (**b**) IMF2 for 500 sampling signals (blue whale vocalization) using CESED.

The CESED of each IMF*i* in the 500 sampled signals was obtained, as shown in Figure 9. The energy distribution of the A call and B call can be clearly observed in Figure 9 for IMF1 and IMF2.

### 2.5. Detection

Through aural listening and visual inspection of the spectrogram or signal energy magnitude, the desired signal and unwanted signal can be manually interpreted to detect the target signal false alarms and missed detections by means of an experienced human operator (EHO) [30]. In the energy distribution function for 500 sampling signals, the average energy of the A call zone was approximately 3.09, whereas the average energy of the B call zone was approximately 21.51. Hence, a threshold of 3.09 was selected. When the signal energy is greater than the threshold, the desired signal is considered to be 1, and when the signal energy is less than the threshold, the signal is regarded as an unwanted signal denoted as 0. Thus, 175 desired signals were obtained from the 500 sampled signals, of which 90 were in the A call zone, 85 were in the B call zone, and 325 were unwanted signals. The detection results generated by the EHO process were considered as real data and compared with the detection data of the detector proposed in this paper. 

In our study, we used the ROC [31] of signal detection theory as a tool to analyze the performance of the detectors, select the best signal detection model, and set the best threshold for the same model. ROC analysis is a two-bit classification model, that is, there are only two categories of detection outputs. In addition, the result of signal detection needs to be defined by an appropriate threshold. Thus, the predicted and true values of four possible parameter results were considered as the number of true positives (TPs), i.e., signals correctly detected as valid, false positives (FPs), i.e., signals incorrectly detected as valid, true negatives (TNs), i.e., signals correctly detected as invalid, and false negatives (FNs), i.e., signals incorrectly detected as invalid. The true-positive rate is defined as TPR = TP/(TP + FN), and the false-positive rate is defined as FPR = FP/(FP + TN). Next, given a two-digit classification model and an appropriate threshold, it is possible to calculate a coordinate point, with the *X*-axis displaying the FPR and the *Y*-axis displaying the TPR, from the true values and predicted values of all sampled signals. Each threshold setting will yield different values for the FPR and TPR. Consider an ROC curve drawn between the X- and Y-axes (0,1). The perfect prediction is at the point (0,1) in the upper left corner of the ROC spatial coordinates, that is, the predicted value and true value of the detection output are 100% concurrent at this point. Therefore, if the curve is closer to the upper left corner of the graph coordinates (0,1), the point above the curve represents a better classification result and the point below the curve represents a poor classification result. Thus, we can obtain the analysis results of the sampling signal detection performance in the ROC curve chart. In our experiment, we evaluated the four signal feature extraction and analysis algorithms described above, ERD, ESD, ESED, and CESED, using 500 sample signals and compared the ROC detection performances. 

In the detection process, according to the Bayes criterion [31], we considered the problem as a binary classification task with two hypotheses: H1 representing the positive class (“yes”) and H0 representing the negative class (“no”). Our goal was to make the optimal classification decision based on the observed feature values. To achieve this, we needed to consider two types of errors: false negatives (positive instances incorrectly classified as negative instances) and false positives (negative instances incorrectly classified as positive instances). By weighting these two types of errors, we were able to choose an appropriate threshold that minimized the classification error.

Specifically, we used a threshold to divide the feature values into two regions: one region representing predictions corresponding to the positive class (“yes”) and the other region representing predictions corresponding to the negative class (“no”). Then, we made the classification decision based on the region where the feature value fell. For example, if the feature value were greater than the threshold, it would be classified as a positive instance; if the feature value were less than the threshold, it would be classified as a negative instance. This paper provides two ways to select thresholds: median and optimal estimated threshold methods.

(1) Based on statistical features (medians): This approach to determining the threshold value utilizes Chebyshev’s inequality theory. According to this theory, the threshold can be set based on the energy of the sampling signal relative to the median plus a certain number of times the deviation is multiplied by a factor M [20]. This method allows for dynamic adjustment of the threshold based on the statistical features of the signal, enabling adaptation to different types of signals. Its advantages include:
  Adaptability: The threshold can be dynamically adjusted based on the statistical features of the signal, allowing it to adapt to variations in different signals;
  Robustness: By considering statistical features, threshold selection becomes more robust with respect to variations in signal characteristics, thereby improving detection performance.
(2) Based on receiver operating characteristic (ROC) analysis (the optimal estimated threshold): Another criterion for selecting the threshold is by analyzing the receiver operating characteristic (ROC) curve. The ROC curve illustrates the trade-off between the true-positive rate and the false-positive rate at different threshold values. The point on the ROC curve closest to the coordinate (0,1) represents the optimal estimated result. Thus, the threshold chosen at this point can be considered the optimal estimated threshold, maximizing the system’s performance in terms of detection accuracy. By describing the process of setting the adaptive threshold based on statistical measures and selecting the optimal estimated threshold using the ROC curve, the study acknowledges the importance of threshold determination and highlights the use of adaptive techniques to enhance the detection accuracy and robustness of the system. Its advantages include:
  Performance optimization: Choosing the threshold based on the ROC curve allows identification of the optimal estimated threshold that maximizes the system’s detection accuracy. This can enhance the overall detection performance;
  Objective evaluation: The ROC curve provides a visual representation of the classifier’s performance, allowing for quantitative assessment of the balance between the true-positive rate and the false-positive rate. This objective evaluation helps in selecting a threshold that balances detection accuracy.

By applying these threshold selection methods, algorithms can effectively determine the appropriate threshold for classifying signals as “yes” or “no,” thereby improving the accuracy and robustness of the detection system. 

## 3. Analysis Results

After processing the 500 sample signals through the four signal feature extraction and analysis algorithms, the signal feature extraction function distribution of each algorithm was obtained, along with the time–energy distribution of ERD, the time–energy distribution of ESD, the energy spectrum entropy distribution of ESED, and the energy spectrum entropy distribution of CESED, as shown in Figure 5, Figure 6, Figure 7 and Figure 8 respectively.

The median values of the IMF1 signal feature extraction function distributions of ERD, ESD, ESED, and CESED were set as the thresholds; these were 25.38, 1.55, 1.69, and 5.45, respectively. The numbers of TPs in the A call zone region were 35, 47, 37, and 47, whereas the numbers of those in the B call zone were 80, 83, 79, and 85, respectively. The sums of the two give the total numbers of detections, which were 115, 130, 116, and 132, respectively. The detection ratios for the TPRs were 65.71%, 74.29%, 66.29%, and 75.43%, respectively. The numbers of FP parameters in the A call zone were 86, 70, 86, and 40, whereas the numbers of those in the B call zone were 50, 38, 47, and 60, respectively. The sums of the two give the numbers of false alarms, which were 136, 108, 133, and 100, respectively, and the FPRs were 41.72%, 33.23%, 40.80%, and 30.37%, respectively, as shown in Table 3.

The median values of the IMF2 signal feature extraction function distributions of ERD, ESD, ESED, and CESED were set as the thresholds; these were 22.42, 3.49, 1.42, and 4.38, respectively. The numbers of TPs in the A call zone were 69, 107, 62, and 72, respectively, whereas the numbers of those in the B call zone were 17, 40, 12, and 85, respectively. The sums of the two give the numbers of detections, which were 86, 107, 74, and 157, respectively. The detection ratios of the TPRs were 49.14%, 61.14%, 42.29%, and 89.71%, respectively. The number of FPs in the A call zone were 96, 77, 97, and 64, respectively, whereas the numbers of those in the B call zone were 73, 56, 86, and 34, respectively. The sums of the two give the numbers of false alarms, which were 169, 133, 183, and 98, respectively, and the FPRs were 52.15%, 40.92% 56.44%, and 30.06%, respectively, as shown in Table 4.

**Figure 10:**
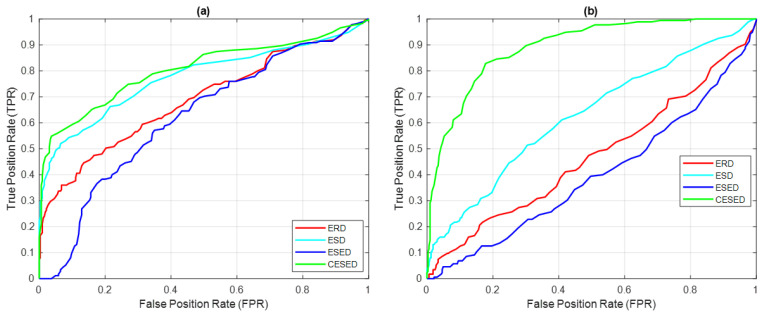
ROCs of ERD, ESD, ESED, and CESED for (**a**) IMF1 and (**b**) IMF2 for 500 sample signals (blue whale vocalization).

Based on these four signal feature extraction function distributions and the threshold settings, the ROC curves for the four detectors in IMF1 and IMF2 were delineated from the highest to the lowest values of the signal feature extraction function distribution, as shown in Figure 10. From these two ROC plots, it is clear that the CESED detector outperformed the other three detectors in terms of signal detection. 

The optimal estimated thresholds of the IMF1 signal feature extraction function distributions of ERD, ESD, ESED, and CESED were 33, 2.4, 1.9, and 5.4, respectively. The numbers of TPs in the A call zone were 24, 28, 31, and 47, whereas the numbers of those in the B call zone were 77, 80, 79, and 85, respectively. The sums of the two give the total numbers of detections, which were 101, 108, 110, and 132, respectively. The detection ratios of the TPRs were 7.71%, 61.71%, 62.86%, and 75.43%, respectively. The numbers of FPs in the A call zone were 73, 45, 78, and 40, respectively, whereas the numbers of those in the B call zone were 27, 17, 35, and 60, respectively. The sums of the two give the numbers of false alarms, which were 100, 62, 113, and 100, respectively, and the FPRs were 30.67%, 19.08%, 34.66%, and 30.37%, respectively, as shown in Table 5. 

**Figure 11:**
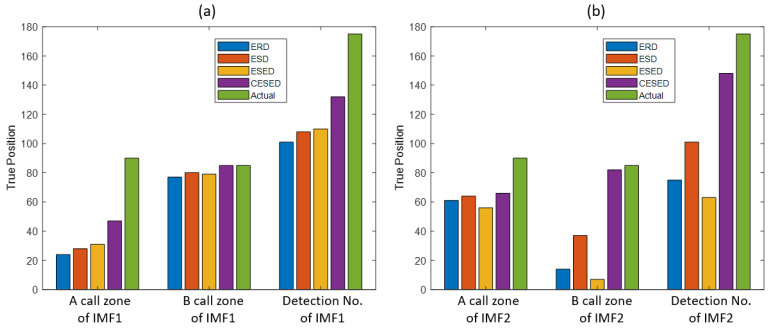
(**a**) The number of TPs in the A call zone of IMF1 and the B call zone of IMF1 and the detection number for IMF1 with ERD, ESD, ESED, and CESED. (**b**) The number of TPs in the A call zone of IMF2 and the B call zone of IMF2 and the detection number for IMF2 with ERD, ESD, ESED, and CESED.

The optimal estimated thresholds of the IMF2 signal feature extraction function distributions of ERD, ESD, ESED, and CESED were 24, 3.8, 1.55, and 4.25, respectively. The number of TPs in the A call zone were 61, 64, 56, and 66, respectively, whereas the numbers of those in the B call zone were 14, 37, 7, and 82, respectively. The sums of the two give the total numbers of detections, which were 75, 101, 63, and 148, respectively. The detection ratios of the TPRs were 42.86%, 57.71%, 36.00%, and 84.57%, respectively. The numbers of FPs in the A call zone were 84, 71, 81, and 46, respectively, whereas the numbers of those in the B call zone were 66, 53, 69, and 23, respectively. The sums of the two give the numbers of false alarms, which were 150, 124, 151, and 69, respectively, and the FPRs were 46.32%, 38.15%, 46.32%, and 21.17%, respectively, as shown in Table 6 and Figure 11. 

In this paper, four detectors (ERD, ESD, ESED, and CESED) were proposed and two different threshold selection methods were used for experimental evaluation: the median and optimal estimated threshold methods. The detailed experimental results are documented in Table 3, Table 4, Table 5 and Table 6. According to the experimental results, CESED exhibited the best detection performance in terms of detection ratio (TPR) and false-alarm ratio (FPR). These results in the paper indicate that the CESED detector outperformed the other detectors in terms of detection performance. Furthermore, choosing the optimal estimated threshold instead of the median threshold yielded better detection performance in the experimental setup.

## 4. Discussion

In ROC curves, the TP parameter represents the number of samples that are positive and correctly predicted as positive by the detector. From the MF distribution, it can be observed that, since the energy of the B call signal was mainly distributed in IMF1, as shown in Figure 11a, the detection abilities of the four signal feature extraction and analysis algorithms in the B call zone of IMF1 were similar, and the TP values are very close to the actual values. However, there was a gap between the TP values and the actual values in the A call zone of IMF1. Regarding the 500 sampled signals of IMF1, the CESED algorithm had the best signal detection ability, and the TPP value reached 75.43%. In addition, Figure 11b shows that, since the energy of the A call signal was mainly distributed in IMF2, the detection abilities of the four signal feature extraction and analysis algorithms in the A call zone of IMF2 were similar, and the TP values were close to each other. However, regarding the B call zone of IMF2, except for CESED, the other three algorithms could not accurately detect the B call signal. Regarding the 500 sampled signals of IMF2, CESED also had the best signal detection ability, with a TPP value of up to 84.57%. Therefore, CESED has good signal detection ability and can successfully detect marine mammal sounds.

The AUC represents the area under the ROC curve and is a statistic that is commonly used to assess the predictive power of a detector. As mentioned earlier, the closer the ROC curve is to the upper left (0,1), the better the predictive ability. Therefore, the larger the area under the ROC curve, the better the predictive power, which means the higher the detection efficiency of the detector. When the AUC is 1, the detector is perfect. When the AUC > 0.5, the detection effect is better than random guessing and the model has a certain predictive value. When the AUC is 0.5, the detection effect of the detector is the same as random guessing and the detector has no predictive value. When the AUC < 0.5, the detector classification effect is worse than random guessing, but if a counter-prediction is made, the detector classification effect can be better than random guessing. 

Accuracy, Precision, Recall, and F1 scores are performance metrics [32] commonly used in machine learning and statistical analysis to evaluate the performance of detection and classification models by calculating ratios based on the four parameters of the ROC curve.

(16) $$Accuracy=\frac{TP+TN}{TP+TN+FP+FN}$$

(17) $$Precison=\frac{TP}{TP+FP}$$

(18) $$Recall\left(Sensitivity\right)=\frac{TP}{TP+FN}$$

(19) $$F1\;Score=2\;*\;\frac{Precision\;*\;Recall}{Precision+Recall}$$

Regarding the 500 sample signals of blue whale vocalization, where the CIMF was selected as IMF2, for the signal feature extraction function distributions of ERD, ESD, ESED, and CESED, the AUCs of the ROC curve were 0.4621, 0.6162, 0.3894, and 0.8979, respectively; the Accuracy scores were 49.90%, 60.40%, 47.50%, and 80.84%, respectively; the Precision scores were 31.19%, 44.89%, 29.44%, and 68.20%, respectively; the Recall scores were 42.83%, 57.71%, 36.00%, and 84.57%, respectively; and the F1 scores were 37.41%, 50.50%, 32.39%, and 75.51%, respectively, based on the thresholds of the optimal estimated results, as shown in Table 7. Further, the four different cetacean sound recordings were acquired from three sources. The sounds of the bowhead whale and the Bryde’s whale were obtained from the website of the Scripps Institution of Oceanography at the University of California, San Diego, CA, USA [33]; the sounds of dolphin whistles were sourced from the supplementary data of reference [21]; the sounds of pattern dolphin clicks were obtained from the website of the Kuroshio Ocean Education Foundation [34]; and the detection performance of the proposed method was evaluated. 

**Figure 12:**
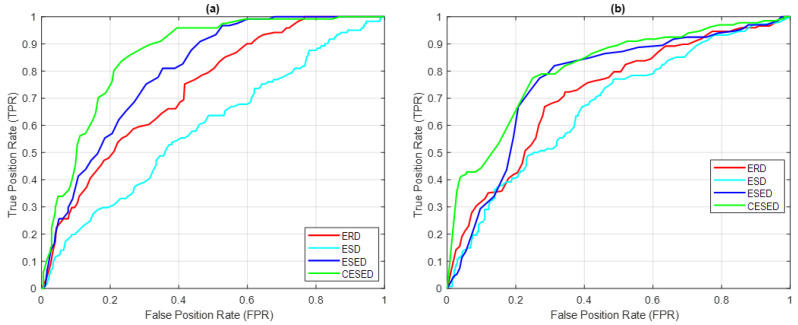
ROC curves for ERD, ESD, ESED, and CESED for the sounds of (**a**) the bowhead whale and (**b**) the Bryde’s whale.

The sound of the bowhead whale had a sampling frequency of 4800 Hz; each signal sampling time was 200 ms, and 500 signals were sampled. The CIMF was IMF2, with an average energy ratio of 34.73%, and the main frequency domain was distributed in the range of 1–100 Hz, as shown in Table 7. In the IMF2 signal feature extraction function distributions of ERD, ESD, ESED, and CESED, the AUCs of the ROC curve were 0.7388, 0.5944, 0.8061, and 0.8980, respectively; the Accuracy scores were 60.61%, 58.99%, 68.89%, and 81.45%, respectively; the Precision scores were 35.77%, 30.84%, 42.53%, and 67.83%, respectively; the Recall scores were 76.86%, 54.55%, 77.69%, and 58.79%, respectively; and the F1 scores were 48.82%, 39.40%, 54.97%, and 80.17%, respectively, based on the thresholds of the optimal estimated results, as shown in Figure 12a. 

The sound of the Bryde’s whale had a sampling frequency of 2400 Hz; each signal sampling time was 200 ms, and 500 signals were sampled. The CIMF was IMF5, the average energy distribution ratio of the CIMF was 29.59%, and the main frequency domain was distributed in the range of 1–100 Hz, as shown in Table 7. In the IMF5 signal feature extraction function distributions of ERD, ESD, ESED, and CESED, the AUCs of the ROC curve were 0.7254, 0.6678, 0.7735, and 0.8320, respectively; the Accuracy scores were 69.60%, 62.55%, 72.99%, and 74.28%, respectively; the Precision scores were 49.02%, 42.61%, 50.48%, and 51.98%, respectively; the Recall scores were 67.57%, 66.22%, 78.95%, and 78.95%, respectively; and the F1 scores were 56.82%, 51.85%, 61.58%, and 62.69%, respectively, based on the thresholds of the optimal estimated results, as shown in Figure 12b. 

The sound of dolphin whistles had a sampling frequency of 96,000 Hz; each signal sampling time was 200 ms, and 200 signals were sampled. The CIMF was IMF1, the average energy distribution ratio of the CIMF was 68.03%, and the main frequency domain was distributed in the range of 2000–8000 Hz, as shown in Table 7. In the IMF1 signal feature extraction function distributions of ERD, ESD, ESED, and CESED, the AUCs of the ROC curve were 0.8800, 0.8945, 0.6777, and 0.7582, respectively; the Accuracy scores were 87.00 %, 86.00%, 61.31%, and 79.00%, respectively; the Precision scores were 44.12%, 42.86%, 18.39%, and 29.17%, respectively; the Recall scores were 68.18%, 81.82%, 72.73%, and 63.64%, respectively; and the F1 scores were 53.57%, 56.25%, 29.36%, and 40.00%, respectively, based on the threshold of the optimal estimated results. 

The sound of pattern dolphin clicks had a sampling frequency of 44,100 Hz; each signal sampling time was 200 ms, and 300 signals were sampled. The CIMF was IMF1, the average energy distribution ratio of the CIMF was 30.96%, and the main frequency domain was distributed in the range of 1–1000 Hz, as shown in Table 7. In the IMF1 signal feature extraction function distributions of ERD, ESD, ESED, and CESED, the AUCs of the ROC curve were 0.7812, 0.6525, 0.7953, and 0.7589, respectively; the Accuracy scores were 67.67%, 58.47%, 75.00%, and 69.44%, respectively; the Precision scores were 51.85%, 43.40%, 62.73%, and 54.35%, respectively; the Recall scores were 81.55%, 66.35%, 66.99%, and 72.12%, respectively; and the F1 scores were 63.40%, 52.47%, 64.79%, and 61.98%, respectively, based on the threshold of the optimal estimated results. The results showed that the CESED algorithm performed the best in detecting marine mammal sounds. Please refer the Table A1 of Appendix A for the descriptions of key features of the four proposed feature extraction algorithms, and the Table A2 of Appendix A for the List and descriptions of all equations featured in the article and their parameters.

## 5. Conclusions

This paper proposed an EMD-based energy spectrum entropy distribution signal detection method for marine mammal vocalizations which involved four signal feature extraction and analysis algorithms: ERD, ESD, ESED, and CESED. The signal feature extraction function distributions, namely, the time–energy distribution of ERD, the time–energy distribution of ESD, the energy spectrum entropy distribution of ESED, and the energy spectrum entropy distribution of CESED, were used to realize sound detection of marine mammals. The analysis of the experimental results showed that the CESED detector performed significantly better than the other three detectors in terms of the AUC and the accuracy of the detection parameters, mainly for the following reasons:

(1) EMD can perform energy decomposition for multicomponent signals in the environment of nonstationary signals and present the energy state of signals as a function of IMFs.
(2) The energy density intensity of the signal was concentrated in the main frequency domain. Energy characteristics analysis and the MF method were used to extract and analyze the signal in the main frequency domain to improve the resolution of the signal analysis.
(3) Theoretical methods of EMD and entropy were used to analyze the parameters of signal data change in the signal feature extraction function distribution and the energy spectrum entropy distribution and achieve the signal detection effect.

In order to develop a diverse passive acoustic monitoring (PAM) system that can adapt to the challenges of complex marine environments, this study utilized the advantages of empirical mode decomposition (EMD) for the analysis of nonstationary signals as well as energy feature analysis and entropy from information theory for the detection of marine mammal vocalizations and the analysis of various marine mammal sound signals. This article proposes a detection method for marine mammal vocalizations using four signal feature extraction analysis algorithms: energy ratio distribution (ERD), energy spectrum distribution (ESD), energy spectrum entropy distribution (ESED), and concentrated energy spectrum entropy distribution (CESED). Among these algorithms, the primary focus was on the innovative research of the concentrated energy spectrum entropy distribution (CESED). By observing the time–energy distribution or the entropy distribution generated by these algorithms, appropriate thresholds were selected based on a threshold-based approach for signal detection using the feature extraction function distribution. The performance of the detection method was evaluated by comparing it with traditional energy ratio analysis and energy spectrum analysis methods, using the performance metrics of AUC, Accuracy, Precision, Recall and F1 score for the ROC curve, and it showed better detection results. This method can be applied in the field of marine mammal sound signal detection theory. Additionally, the establishment of a complete cetacean bioacoustics database could be a target of future efforts. By applying the proposed detection method to actual preprocessed whale and dolphin sounds, the analysis and detection of signal features from sampled audio signals can be performed. This can serve as a crucial technology for the development of marine bioacoustics monitoring systems, marine biology research, and defense technology. 

## Figures and Tables

**Table 1:** Average energy ratio of each IMF*i* for 500 sampling signals (blue whale vocalization).

IMF*i*	IMF1	IMF2	IMF3	IMF4	IMF5	IMF6	IMF7
Average energy ratio (%)	31.92	25.09	19.29	12.14	6.62	1.90	0.21

**Table 2:** Signal extraction parameters of 500 sampling signals (blue whale vocalization).

IMF*i*	Threshold	Frequency of Highest Energy Ratio (Hz)	Main Frequnecy Band (Hz)	Main Frequency Domain (Hz)
IMF1	0.3	39	27~49	1~150
IMF2	0.3	13	9~43	1~100

**Table 3:** IMF1 signal feature extraction function distributions of ERD, ESD, ESED, and CESED with the parameters TP and FP, with the medium as the threshold.

IMF1 Threshold (Median)	True Position (TP)				False Position (FP)			
	A Call Zone Number	B Call Zone Number	Detection Number	Detection Ratio (TPR)	A Call Zone Number	B Call Zone Number	False Alarm Number	False Alarm Ratio (FPR)
ERD	35	80	115	65.71%	86	50	136	41.72%
ESD	47	83	130	74.29%	70	38	108	33.23%
ESED	37	79	116	66.29%	86	47	133	40.80%
CESED	47	85	132	75.43%	40	60	100	30.37%

**Table 4:** IMF2 signal feature extraction function distributions of the ERD, ESD, ESED, and CESED with the parameters TP and FP, with the median as the threshold.

IMF2 Threshold (Median)	True Position (TP)				False Position (FP)			
	A Call Zone Number	B Call Zone Number	Detection Number	Detection Ratio (TPR)	A Call Zone Number	B Call Zone Number	False Alarm Number	False Alarm Ratio (FPR)
ERD	69	17	86	49.14%	96	73	169	52.15%
ESD	107	40	107	61.14%	77	56	133	40.92%
ESED	62	12	74	42.29%	97	86	183	56.44%
CESED	72	85	157	89.71%	64	34	98	30.66%

**Table 5:** IMF1 signal feature extraction function distributions of ERD, ESD, ESED, and CESED with the parameters TP and FP and the optimal estimated thresholds.

IMF1 (the Optimal Estimated Threshold)	True Position (TP)				False Position (FP)			
	A Call Zone Number	B Call Zone Number	Detection Number	Detection Ratio (TPR)	A Call Zone Number	B Call Zone Number	False Alarm Number	False Alarm Ratio (FPR)
ERD	24	77	101	57.71%	73	27	100	30.67%
ESD	28	80	108	61.71%	45	17	62	19.08%
ESED	31	79	110	62.86%	78	35	113	34.66%
CESED	47	85	132	75.43%	40	60	100	30.37%

**Table 6:** IMF2 signal feature extraction function distributions of ERD, ESD, ESED, and CESED with the parameters TP and FP and the optimal estimated threshold.

IMF2 (the Optimal Estimated Threshold)	True Position (TP)				False Position (FP)			
	A Call Zone Number	B Call Zone Number	Detection Number	Detection Ratio (TPR)	A Call Zone Number	B Call Zone Number	False Alarm Number	False Alarm Ratio (FPR)
ERD	61	14	75	42.86%	84	66	150	46.32%
ESD	64	37	101	57.71%	71	53	124	38.15%
ESED	56	7	63	36.00%	81	69	151	46.32%
CESED	66	82	148	84.57%	46	23	69	21.17%

**Table 7:** Performance metrics of ERD, ESD, ESED, and CESED for AUC, Accuracy, Precision, Recall, and F1 scores of CIMFs with the optimal estimated thresholds for the blue whale, the bowhead whale, the Bryde’s whale, dolphin whistles, and pattern dolphin clicks, along with key parameters, including sampling frequency, sampling time, number of samples, average energy ratio, and main frequency domain.

Species	Sampling Frequency (Hz)	Sampling Time (ms)	Number of Sampled	CIMF	Average Energy Ratio	Main Frequency Domain (Hz)	Optimal Estimated Threshold	Performance Metric	ERD	ESD	ESED	CESED
Blue whale [27]	4800	200	500	2	25.09%	1~100	4.38	AUC Accuracy Precision Recall F1 score	0.4621 49.90% 31.19% 42.83% 37.41%	0.6162 60.40% 44.89% 57.71% 50.50%	0.3894 47.50% 29.44% 36.00% 32.39%	0.8979 80.84% 68.20% 84.57% 84.57%
Bowhead whale [29]	4800	200	500	2	34.73%	1~100	2.55	AUC Accuracy Precision Recall F1 score	0.7388 60.61% 35.77% 76.86% 48.82%	0.5944 58.99% 30.84% 54.55% 39.40%	0.8061 68.89% 42.53% 77.69% 54.97%	0.8980 81.45% 67.83% 58.79% 80.17%
Bryde’s whale [29]	2400	200	500	5	29.59%	1~100	1.10	AUC Accuracy Precision Recall F1 score	0.7254 69.60% 49.02% 67.57% 56.82%	0.6678 62.55% 42.61% 66.22% 51.85%	0.7735 72.99% 50.48% 78.95% 61.58%	0.8320 74.28% 51.98% 78.95% 62.69%
Dolphin whistle [31]	96,000	200	200	1	68.03%	2000~8000	8.36	AUC Accuracy Precision Recall F1 score	0.8800 87.00% 44.12% 68.18% 53.57%	0.8945 86.00% 42.86% 81.82% 56.25%	0.6777 61.31% 18.39% 72.73% 29.36%	0.7582 79.00% 29.17% 63.64% 40.00%
Pattern dolphin click [32]	44,100	200	300	1	30.96%	1~1000	7.10	AUC Accuracy Precision Recall F1 score	0.7812 67.67% 51.85% 81.55% 63.40%	0.6525 58.47% 43.40% 66.35% 52.47%	0.7953 75.00% 62.73% 66.99% 64.79%	0.7589 69.44% 54.35% 72.12% 61.98%

## Data Availability

Not applicable.

## Abbreviations

AUC	Area under the curve
CESED	Concentrated energy spectrum entropy distribution
CIMF	Competent intrinsic mode function
EHO	Experienced human operator
EMD	Empirical mode decomposition
ESD	Energy spectrum distribution
ESED	Energy spectrum entropy distribution
ERD	Energy ratio distribution
FN	False negative
FP	False positive
HHT	Hilbert–Huang transform
HMM	Hidden Markov model
IF	Instantaneous frequency
IMF	Intrinsic mode function
MBARI	Monterey Bay Aquarium Research Institute
MF	Marginal frequency
MFCCS	Mel-scale frequency cepstral coefficients
NN	Neural network
PAM	Passive acoustic monitoring
*rf*	Residual function
ROC	Receiver operating characteristics
SE	Sample entropy
STFT	Short-time Fourier transform
SVM	Support vector machine
TFD	Time–frequency distribution
TN	True negative
TP	True positive
WT	Wavelet transform

## Appendix A

**Table A1:** Descriptions of key features of the four proposed feature extraction algorithms.

Algorithms	Key Features		Distribution
ERD	E*_IMFi_*(*t*)	The energy ratio of IMF*i* with the total energy	Time-energy
ESD	Max(E*_IMFi_*(*f*))	Highest energy of spectrum with the main frequency domain (*f = f_1_~f_2_*)	Time-energy
ESED	Hs*i* E*ij* P(E*ij*) H*_i_*(E)	Hilbert Spectrum of IMF*i* Energy distribution, *i* is the IMFs, j is the frequency(*m~n*) Probability functions Energy spectrum entropy with the total energy	Time-entropy
CESED	CH*_i_*(E)	Energy spectrum entropy with the main frequency domain (*f = f_1_~f_2_*)	Time-entropy

**Table A2:** List and descriptions of all equations featured in the article and their parameters.

Index	Equation	Description	Parameters	Description
1	$X\left(t\right)=\sum_{i=1}^{N}{IMF}_{i}\left(t\right)+rf\left(t\right)$	The EMD method decomposes a signal into a set of IMFs and an *rf*	IMF*i*(t)	The *i*th intrinsic mode function
			*rf*(t)	The residual function
2	$E_{total}=\sum_{i=1}^{N}{IMF}_{i}^{2}\left(t\right)+{rf}^{2}\left(t\right)$	The total energy is the sum of the energies of all the IMFs and *rf*	E_total_	The total energy of the signal
3	$E_{IMFi}=\frac{{IMF}_{i}^{2}}{E_{total}}\;*\;100\%$	The *i*th IMF energy ratio is divided by the total energy, E_total_	E_IMF*i*_	The *i*th IMF energy ratio
4	$X_{i}\left(t\right)={IMF}_{i}\left(t\right)+jHT\left\{{IMF}_{i}\left(t\right)\right\}=A_{i}\left(t\right)e^{j\theta_{i}\left(t\right)}$	The signal can be expressed as the sum of the real and imaginary parts	HT{IMF*i*(t)}	Hilbert transform for the *i*th intrinsic mode function
			A*i*(t)	Amplitude of signal
			*θi*(t)	Angular frequency of signal
5	$F_{i}\left(t\right)=\frac{1}{2\pi }\frac{d\theta_{i}\left(t\right)}{dt}$	By taking the derivative of the phase angle and dividing it by 2π, the instantaneous frequency of the IMF can be obtained	F*i*(t)	Instantaneous frequency
6	${MF}_{i}=\frac{{IMF}_{if}^{2}\left(t\right)}{E_{total}}\;*\;100\%$	The sampling frequency bandwidth is f Hz, the MF (frequency–energy distribution) of the ith IMF	MF*i*	The *i*th IMF marginal frequency distribution
7	$max(E_{IMFi}(f))=max\left\{{MF}_{i}(f)|f=f_{1}\sim f_{2}\right\}$	The energy of all instantaneous frequencies is scanned and the frequency of the highest energy ratio in the main frequency domain of MF; f: f1–f2 is the main frequency domain of the MF of the sampling signal		
8	${HS}_{i}=\frac{E_{i}\left(t,f\right)}{E_{total}}=\frac{{IMF}_{i}^{2}\left(t,f\right)}{E_{total}}$	Hilbert energy spectrum (HS; time–frequency–energy distribution)	E*i*(*t*,*f*)	The energy distribution function Ei(t, f) contains the sampling time (t) and the sampling frequency (f)
9	$P_{i}\left(E_{j}\right)=P\left(E_{ij}\right)=\frac{E_{ij}}{E_{total}}$	The energy distribution function can be expressed by the probability functions; it is normalized according to the total energy of the signal	P(E*ij*)	The probability functions
			E*ij*	The energy distribution, *i* is the IMF number, and *j* is the sampling frequency range m to n
10	$ESED=H\left(E\right)=-\sum_{i=1}^{i}\sum_{j=m}^{n}P\left(E_{ij}\right)\log P\left(E_{ij}\right)$	The entropy of the energy spectrum of each sampling signal	H(E)	Energy spectrum entropy distribution (ESED)
11	$ESED\;of\;ith\;IMF=H_{i}\left(E\right)=-\sum_{j=m}^{n}P_{i}(E_{j})\log P_{i}(E_{j})$	The energy spectrum entropy (H) of each IMFi can also be determined		
12	$H_{icd}\left(E\right)=-\sum_{j=c}^{d}P_{i}(E_{j})\log P_{i}(E_{j})$	The energy spectrum entropy of each IMFi in the main frequency domain, called H*icd*, and the main frequency domain, which ranges from c to d		
13	$S_{ij}=\frac{E_{ij}-\min\left(E_{ij}\right)}{\max\left(E_{ij}\right)-\min\left(E_{ij}\right)}$	S*ij*, the signal energy density of the ith IMF in the sampling signal, where i is the IMF number of the sampling signal and j is the main frequency domain from a to b		
14	$P_{i}\left(E_{j}\right)=P\left(E_{ij}\right)=\frac{S_{ij}}{\sum_{j=a}^{b}S_{ij}}$	The energy distribution function can be expressed by the probability functions; it is normalized according to the main frequency domain of the sampling signal		
15	$CESED\;of\;the\;ith\;IMF={CH}_{i}\left(E\right)=-\sum_{j=a}^{b}P_{i}(E_{j})\log P_{i}(E_{j})$	The concentrated energy spectrum entropy (CH) of each IMFi can also be determined		
